# e-Delphi study on European expert views about “cancer survivor” and related terminologies

**DOI:** 10.1007/s11764-026-01972-z

**Published:** 2026-01-31

**Authors:** Amy Taylor, Andrew Davies, PRiSM (Specialist Palliative Care’s Role in Cancer Survivorship Model) e-Delphi Group

**Affiliations:** 1 School of Medicine, Trinity College Dublin, Dublin, Ireland; 2 Academic Department of Palliative Medicine, Our Lady’s Hospice & Care Services, Harold’s Cross, Dublin, D6W RY72 Ireland; 3 School of Medicine, University College Dublin, Dublin, Ireland

**Keywords:** Cancer survivors, Cancer survivorship, Nomenclature, Living beyond cancer

## Abstract

**Purpose:**

Definitions of “cancer survivors” vary from individuals from diagnosis throughout their life, to individuals post-curative intent treatment. Attempts to categorise groups of people who have or had cancer also differ. This study aims to generate European expert consensus opinion about cancer survivor terminology and categorisation relevant to developing a model of care.

**Methods:**

This was an electronic Delphi study where specialist palliative care (SPC) and oncology (ONC) European experts, including patient advocates, were purposively sampled to form two separate groups. Participants selected agreement with 105 questions/statements developed from literature reviews in round 1, and in subsequent rounds statements were modified/added according to feedback. The study was terminated when consensus (pre-defined as ≥ 75% within each group) and stability (determined by Spearman’s Rank Order Correlation) were reached for most statements. The Chi square test for goodness of fit compared agreement between groups.

**Results:**

Eighty-six SPC experts from 19 countries/regions, and 54 ONC experts from 17 countries/regions began the process, with three rounds completed by 80 and 49 respectively. Eight statements about “cancer survivor” application reached consensus in both groups, demonstrating agreement that “cancer survivor” should be applied to people post-treatment with no evidence of disease. Two other statements reached consensus in only one group (with a significant difference). Two categorisations reached consensus in both groups.

**Conclusions:**

These experts suggest that application of “cancer survivor” to people with active cancer should be reconsidered.

**Implications for Cancer Survivors:**

Pragmatic categorisation of people who have/had cancer could help provide tailored care for different groups.

**Supplementary Information:**

The online version contains supplementary material available at https://doi.org/10.1007/s11764-026-01972-z.

## Introduction

Cancer survivor terminology is extremely contentious [1]. The most widely accepted definition from the National Cancer Institute (NCI) [2] states that, “an individual is considered a cancer survivor from the time of diagnosis through the balance of life. There are many types of survivors, including those living with cancer and those free of cancer”. Although many organisations endorse this definition [3, 4], a number of alternate functional definitions have been adopted in clinical practice. For example, the European Society for Medical Oncology (ESMO) [3, p.1121] uses “patients with a diagnosis of cancer who are managed with curative intent, from completion of active therapy (despite eventually on or off maintenance therapy) to long-term follow-up”. However, upon its establishment the *Journal of Cancer Survivorship* highlighted the challenge of identifying the end of primary treatment in such a description, stating that some may have completed “the major aspects of treatment and either desire or need to ‘get on with their lives’” [5, p.7]. Consequently, the Journal noted the importance of contributors explicitly defining their study population, and that the definition of cancer survivors would likely continue to evolve [5].

It has been suggested that the term “cancer survivor” should not be routinely utilised, since not every person with cancer will want to be ascribed a survivor, especially given its everyday connotation (i.e. that the related event/episode has ended) [2, 6]. Many studies have sought the views of people with a history of cancer about this terminology, and these have identified a breadth of interpretations and endorsements [1]. However, most studies have excluded people on anticancer treatment (with an uncertain prognosis), and especially people with advanced/progressive disease (with a limited prognosis). Intuitively, these groups may be more reticent about this terminology. Given its American origins, first proposed by the National Coalition for Cancer Survivorship (a cancer advocacy organisation) [7], it has also been suggested that support for the terminology may be less outside of North America [8]. Despite these concerns, cancer survivor terminology continues to be widely applied within the domains of education, research, and clinical practice.

“Cancer survivorship” is conceptually different from “cancer survivors” (although often used interchangeably), and refers to the physical, psychological, social and economic issues affecting an individual with a history of cancer [9]. The Institute of Medicine states that the essential components of survivorship care include prevention (of late effects and second/recurrent cancers), surveillance (for late effects and cancer), intervention (for consequences of cancer and its treatment) and care coordination [10]. Notably, the Institute of Medicine describes cancer survivorship as “the period following first diagnosis and treatment and prior to the development of a recurrence…or death” [10, p.23]. However, others adopt different criteria, with the Multinational Association of Supportive Care in Cancer (MASCC) suggesting that survivorship spans the time between diagnosis and the end of life (in line with the NCI definition of cancer survivor) [11]. Accordingly, recent literature commonly applies cancer survivorship to people following a cancer diagnosis [12], and indeed standards and practice recommendations for quality survivorship care for people affected by advanced or metastatic cancer have been developed [13].

As well as semantics, there is the issue that while all people with cancer are different, the problems encountered by individuals with early cancer are somewhat different from those seen in people with advanced cancer, and again somewhat different from individuals “free of cancer”. For example, chronic gastrointestinal problems caused by anticancer treatments require specialist interventions, such as rectal bleeding from radiation-induced telangiectasia [14]. Furthermore, interventions that may be entirely appropriate to manage those with active cancer may be less appropriate in people with no active cancer, e.g. long-term opioid analgesics for chronic pain control will have different risk–benefit considerations for someone with advanced progressive cancer and someone with no active cancer [15]. Thus, from a clinical (and research) perspective, there is an imperative to categorise people with cancer or a history of cancer. Additionally, this will support the development of appropriate/tailored services for these various cohorts of people.

Mullan [16] described cancer survivorship as three phases: (1) “acute survival” [p.271], encompassing diagnosis and treatment; (2) “extended survival” [p.272], upon completion of treatment, including surveillance; and (3) “permanent survival” [p.272] following cure. Updates to this model have been suggested as the complexity of people who have/had cancer has evolved. Miller et al. [17] introduced the “transitional” phase (from active treatment to observation), and further detail was added to the “extended” and “permanent” phases [17, p.369]. Subsequently, an additional “chronic” phase (living with cancer for a prolonged time) [18, p.627], and an “end of life” phase were proposed [19]. The University of Texas/MD Anderson Cancer Center [20, p.1] developed the “living with, through and beyond cancer” terminology, which relates to on-treatment, post-treatment and long-term survivorship phases. This categorisation is popular, although there remains some ambiguity about these three phases.

Given the diversity of interpretations, applications and endorsement of “cancer survivor”, and the variety and complexity of people who have or had cancer, there is benefit in clarifying its application, and in identifying groups of people who have or had cancer, particularly in relation to service provision. The PRiSM Project (Specialist Palliative Care’s Role in Cancer Survivorship Model) aims to develop European expert consensus about the role of specialist palliative care for people who have or had cancer. This article presents results from the project relating to use of the term “cancer survivor”, existing categorisations, and a novel categorisation developed to support the model.

## Methods

### Design

This project was an electronic Delphi (e-Delphi) study undertaken via the online platform Welphi® (Lisbon, Portugal) [21] from April to June 2024. The study employed a modified Delphi technique where two simultaneous e-Delphi processes were undertaken, each with European expert groups: one from specialist palliative care (SPC) and the other from oncology (ONC). Delphi methodology is appropriate to build consensus around a controversial issue where there are diverse opinions [22]. The study is reported according to the Guidance on Conducting and REporting DElphi Studies (CREDES) in palliative care [22].

### Expert panels

Participants required expertise in SPC or ONC, English language proficiency and access to the online platform. Criteria for expert selection included: individuals with leadership roles in relevant international specialist palliative care or oncology professional and non-professional organisations (and associated national organisations/member societies); individuals highlighted from a background literature review that focused on the role of specialist palliative care services in cancer survivorship [15]; individuals with European expertise in specialist palliative care and/or oncology in clinical and/or academic and/or other relevant settings across disciplines, particularly cancer survivorship; or individuals recommended by those meeting previous criteria.

Relevant professional organisations, Patient and Public Involvement (PPI) groups and patient advocacy groups (with an interest in cancer survivorship) were invited to nominate individuals within the criteria, the first or corresponding author was identified from articles in the literature review [15], and other experts were contacted personally (purposive sampling). Participants were also asked to suggest other individuals with relevant expertise (snowball sampling). Potential participants were provided with study information via email, and asked to return a study-specific Consent Form.

### Preparatory phase

Two literature reviews were undertaken. In one review, four electronic databases (Medline, Embase, CINAHL, PsycInfo) and websites of leading specialist palliative care and oncology organisations were searched, and information relating to the expertise of people living beyond cancer was identified from all types of relevant articles in the English language [15]. In the second review, another search strategy was applied to the same databases, and data was extracted from original articles published in English about the definition or categorisation of cancer survivors. These reviews were used to inform development of 105 questions/statements for Round 1 (developed by AT and AD, with feedback from members of an Academic Department of Palliative Medicine (ADPM) in Ireland). A pilot round was then undertaken with a specialist palliative care multidisciplinary team from Ireland via the Welphi® platform [21], leading to changes to enhance clarity and usability.

### Delphi rounds

Participants provided basic demographic details, and anonymous feedback on relevant statements over three rounds in order to generate consensus wherever possible. Participants were asked to rate their level of agreement with statements using a five-part Likert scale (i.e. “strongly disagree”, “moderately disagree”, “neither agree nor disagree”, “moderately agree”, “strongly agree”). They were also given the opportunity to provide comments about modifying the statements, and adding new statements. Qualitative content analysis of these responses was undertaken by the two researchers (AT and AD) following every round, informing questionnaire changes in subsequent rounds.

Rounds 2 and 3 included both original statements, and new/modified statements. Anonymous group results were shared with the same group’s participants in these rounds (but not the other group) [23, 24]. Questions that had achieved stable agreement were excluded from subsequent rounds [25]. Each round was open for two weeks, with one week between rounds. Participants were emailed personalised links to the platform at the beginning of each round, reminded about approaching deadlines, and notified upon round closure. The process was terminated after three rounds, when stable consensus had been achieved for the majority of statements (see data analysis) [26].

### Questionnaire content

Specifically, the participants were asked about their views on the NCI definition of cancer survivor (see above) [2], the ESMO definition of cancer survivor (see above) [3], which of these two definitions they preferred, and whether there was an alternative definition they preferred. In addition, participants were asked about their views on who should be called a cancer survivor with options relating to disease status, treatment status, and expectation of treatment. Participants were also asked about their views on the “living with, through and beyond cancer” categorisation [20, p.1], a novel ADPM categorisation developed by the researchers and refined by participants (Box 1), and the so-called “seasons of survivorship” (Box 2) [17].

Box 1: Categorisation of people who have or had cancer.

**Table:** 

**Living with, through and beyond cancer** categorisation [20]:
• Living with cancer—Begins with diagnosis. The focus is on treatment, getting rid of the cancer, if possible, or controlling the cancer to the point where you can live with it long term
• Living through cancer—The period after treatment when you are at risk of your cancer returning (recurrence). This is also a time of rehabilitation
• Living beyond cancer—Post-treatment and long-term survivorship
**ADPM categorisation of people who have or had cancer**:
• Individuals with cancer before the start of primary anticancer treatment
• Individuals with cancer on anticancer treatment with “curative intent” (primary or other)
• Individuals with cancer on anticancer treatment with “palliative”/non-curative intent (primary or other)
• Individuals with cancer not on anticancer treatment on “watch and wait”/active monitoring
• Individuals with cancer not on anticancer treatment with advanced/progressive disease and no further anticancer treatment options
• Individuals with a history of cancer who have completed anticancer treatment and have no evidence of disease, on active oncology follow-up (some risk of recurrence)
• Individuals with a history of cancer who have completed anticancer treatment and have no evidence of disease, not on active oncology follow-up (low risk of recurrence)

Box 2: Seasons of survivorship [16]

**Table:** 

**Seasons of survivorship** [16, p.369]:
• Acute survivorship—Diagnosis, staging and treatment
• Transitional survivorship—The transition from active treatment to careful observation and the emotional, social, and medical adaptations that occur
• Extended survivorship—A diverse group who are who are (1) alive and “living with cancer” but requiring ongoing treatment for recurrent, active, and often advanced disease; (2) in a complete remission that requires ongoing therapy; or (3) in a complete remission and with a favourable prognosis
• Permanent survivorship—A diverse group of 4 subgroups including (1) survivors who are “cancer-free but not free of cancer,” (2) survivors who are cancer-free but continue to have significant “fall-out” from cancer and its treatment including psychosocial, medical, financial, or legal sequelae, (3) survivors who go on to develop second cancers which may be unrelated to the first cancer or its treatment, or may be more likely due to genetic or environmental factors, and (4) survivors who later develop cancers that are secondary to the initial treatment

### Data analysis

Descriptive statistics were used for age, sex, country of residence and professional discipline. Crude figures of response, completion and drop-out rates were recorded for each round. Frequencies of answers (percentages) for each question and statement were determined via the Welphi® platform [21]. Consensus was pre-defined as ≥ 75% having either moderate/strong agreement, moderate/strong disagreement, or neutrality [27, 28]. Stability of levels of agreement with each statement across two rounds was determined using Spearman’s Rank Order Correlation (rho) [26]. Values of 0.80–1.00 represent a very strong association, 0.50–0.80 a moderate association, 0.20–0.5 a fair association, and < 0.2 a poor association [29]. The Chi-square (χ2) test for goodness of fit was used to compare final results from the two expert groups. Statistical tests were carried out using Statistical Package for the Social Sciences (SPSS) version 29.0.2.0.

### Ethical issues

Ethical approval was received from Trinity College Dublin Faculty of Health Sciences Research Ethics Committee (project number 2786), and the study was registered on the Comet Initiative database (https://www.comet-initiative.org/Studies/Details/3097). Participants provided written informed consent. All responses were anonymised from the researchers via the Welphi® platform [21], and all data was treated as confidential.

## Results

Sixty-two professional organisations were invited to recommend participants. Five international organisations and 18 national organisations responded. Fifteen PPI/patient advocacy groups were also invited, and three responded. The authors from four relevant journal articles were contacted, and four authors responded. Other participants were recommended by those who consented. Ninety SPC experts agreed to participate, 86 completed consent, representing 19 countries/regions, and 80 completed all three rounds. Sixty-one ONC experts agreed to participate, 54 completed consent, representing 17 countries/regions, with 49 completing all three rounds. Participant demographics are presented in Table 1, and Table 2 shows retention rates across rounds. Each group included professional experts from a range of disciples and non-professional experts which included patient advocates and people with lived experience.

**Table 1:** E-Delphi participant demographics

**Characteristic**	**Specialist palliative care expert group** (*n* = 86)	**Oncology expert group** (*n* = 54)
**Sex** *n* (%)	Female—45 (52%) Male—41 (48%)	Female—31 (57.5%) Male—23 (42.5%)
**Age**	Median – 52 yr (range: 33–68 yr)	Median – 50 yr (range: 31–70 yr)
**Professional/non-professional** *n*(%)	Professional 82 (95%) Non-professional (PPI group) 4 (5%)	Professional 49 (91%) Non-professional (Patient advocacy groups) 5 (9%)
**Country/region of residence** *n (*%)	Austria 3 (3%) Belgium 1 (1%) Denmark 6 (7%) France 5 (6%) Germany 6 (7%) Greece 1 (1%) Ireland 15 (17%) Italy 3 (3%) Malta 2 (3%) Netherlands 2 (3%) Norway 5 (6%) Poland 6 (7%) Portugal 4 (5%) Romania 2 (3%) Serbia 1 (1%) Spain 5 (6%) Sweden 3 (3%) Switzerland 3 (3%) UK 13 (15%)	Belgium 1 (2%) Cyprus 1 (2%) Denmark 1 (2%) France 4 (7%) Germany 1 (2%) Ireland 6 (11%) Italy 6 (11%) Netherlands 6 (11%) Norway 3 (6%) Poland 1 (2%) Portugal 4 (7%) Serbia 1 (2%) Slovenia 1 (2%) Spain 2 (4%) Sweden 2 (4%) Switzerland 3 (6%) UK 11 (19%)
**Discipline (professional experts)** *n* (%)	Dietetics 1 (1%) Medical sciences 1 (1%) Medicine 59 (69%) Nursing 16 (19%) Occupational therapy 1 (1%) Pharmacy 2 (2%) Psychology 1 (1%) Management 1 (1%)	Medicine 36 (66%) Nursing 8 (15%) Pharmacy 1 (2%) Physiotherapy 2 (4%) Psychology 1 (2%) Skin therapist 1 (2%)

**Table 2:** Retention rates across rounds

		Round 1 *n* (%)	Round 2 *n* (%)	Round 3 *n* (%)
Number of participants completing/number of participants approached	Specialist palliative care expert group	84/86 (98%)	81/84 (96%)	80/81 (99%)
	Oncology expert group	53/54 (98%)	52/53 (98%)	49/52 (94%)
Reason for attrition		No response—3	No response—2 Leave—1 IT issues—1	No response—3 Time commitment—1

In round 1, the SPC participants made 76 comments, and the ONC participants 53 comments. As a result, one statement was divided into two new statements, two statements about known definitions were added, and one categorisation was modified for round 2. In round 2, the SPC participants made a further eight comments, and the ONC participants a further seven comments. Only two statements appeared in round 3 as the remainder had already achieved stable agreement.

Overall, there were thirteen relevant statements in round 1. Six statements reached consensus in the SPC group and seven in the ONC group. There were sixteen relevant statements in round 2, and nine reached consensus in the SPC group and ten in the ONC group. In the final round, both of the two relevant statements reached consensus in the SPC group and one reached consensus in the ONC group. Overall, ten statements reached consensus in both groups and two other statements in only one of the groups.

### Definitions of “cancer survivor”

Consensus was achieved in both expert groups regarding the ESMO definition [2], but not the NCI definition [1] (Table 3). Moreover, the majority in both groups stated that the ESMO definition was their preferred definition (i.e. 54.8% SPC group, 50.9% ONC group). Only 22 (26.2%) of the SPC group and 19 (35.9%) of the ONC group selected the NCI definition as their preference. Six (7.1%) of the SPC group, and three (5.7%) of the ONC group, preferred an alternative definition. However, 10 (11.9%) of the SPC group, and four (7.5%) of the ONC group preferred no definition.

**Table 3:** Expert groups’ agreement with standard definitions of “cancer survivor”

**Definition**	**Group**	Strongly disagree	Moderately disagree	Neither agree nor disagree	Moderately agree	Strongly agree
NCI definition [2]	Specialist palliative care expert group (%)	26	18	13	23	21
		44		13	44	
	Oncology expert group (%)	12	19	12	17	40
		31		12	57	
ESMO definition [3]	Specialist palliative care expert group (%)	1	6	10	43	40
		7		10	83_a_	
	Oncology expert group (%)	6	8	6	40	40
		14		6	80_a_	

^a^ Consensus reached (≥ 75%)

### Subtypes of “cancer survivor”

Table 4 shows the statements about who should be called a cancer survivor that reached consensus in both expert groups (*n* = 7), the SPC group only (*n* = 1), and the ONC group only (*n* = 1). The statements that did not reach consensus were (a) individuals who are still receiving anticancer treatment; (b) individuals who have just completed anticancer treatment, and still have some known cancer present; and (c) no individual should be called a “cancer survivor”, i.e. the term should not be used (see supplementary information, Tables S1a, S2a). Sixteen percent of the SPC group, and 14% of the ONC group, agreed with the latter statement.

**Table 4:** Expert groups’ agreement about who should be called a “cancer survivor” and categorisations of individuals with cancer or a history of cancer

Statement	% SPC group	% ONC group	Agreement between groups
***Statements about who should be called a cancer survivor***			
**Expert group agrees that the following groups should be called cancer survivors:**			
Individuals who have just completed anticancer treatment, and have no known cancer present	75	83	Χ^2^ = 2.000; *p* = *0.157*
Individuals who completed anticancer treatment at least 1 year beforehand, and have no known cancer present	73_a_	89	Χ^2^ = 6.441; *p* = 0.011*
Individuals who completed anticancer treatment at least 5 years beforehand, and have no known cancer present	90	95	Χ^2^ = 0.995; *p* = *0.319*
Individuals who completed anticancer treatment at least 10 years beforehand, and have no known cancer present	88	96	Χ^2^ = 2.773; *p* = *0.096*
**Expert group disagrees with statements that the following groups should be called cancer survivors:**			
All individuals with cancer	82	79	Χ^2^ = 0.294; *p* = *0.588*
Individuals who are still receiving anticancer treatment with curative intent	77	53_a_	Χ^2^ = 49.133; *p* < 0.001*
Individuals who are still receiving anticancer treatment with palliative intent	85	77	Χ^2^ = 16.641; *p* < 0.001*
Individuals who have progressive/worsening cancer, and have no other anticancer treatment options	87	87	Χ^2^ = 1.300; *p* = *0.254*
Family members, friends, and caregivers of individuals with cancer or a history of cancer	94	89	Χ^2^ = 3.071; *p* = *0.080*
***Categorisations of individuals with cancer or a history of cancer***			
**Expert group agrees that the following categorisations are useful:**			
Living with, through and beyond cancer [14]	84	91	Χ^2^ = 1.577; *p* = *0.209*
Novel ADPM categorisation for people who have or had cancer (see Box 1)	89	79	Χ^2^ = 5.327; *p* = 0.021*

^a^ Group proportion was subthreshold 75%, i.e. consensus was not reached in that expert group

* *p* value was significant at a level of < 0.05 Chi square (χ2) test for goodness of fit for one degree of freedom comparing agreement between groups

### Categories of individuals with cancer or a history of cancer

Consensus was also achieved in both expert groups that the living with, through and beyond cancer categorisation, and the novel ADPM categorisation would be useful to inform a model of care (Table 4). However, consensus was not achieved in either expert group about the so-called seasons of cancer survivorship, with a majority (65%) of SPC group disagreeing with the categorisation, and a slight majority (47%) of ONC group agreeing with the categorisation (X^2^ (1) = 40.540; *p* < **0.001**) (see supplementary information, Tables S1b, S2b).

## Discussion

**Fig. 1:**
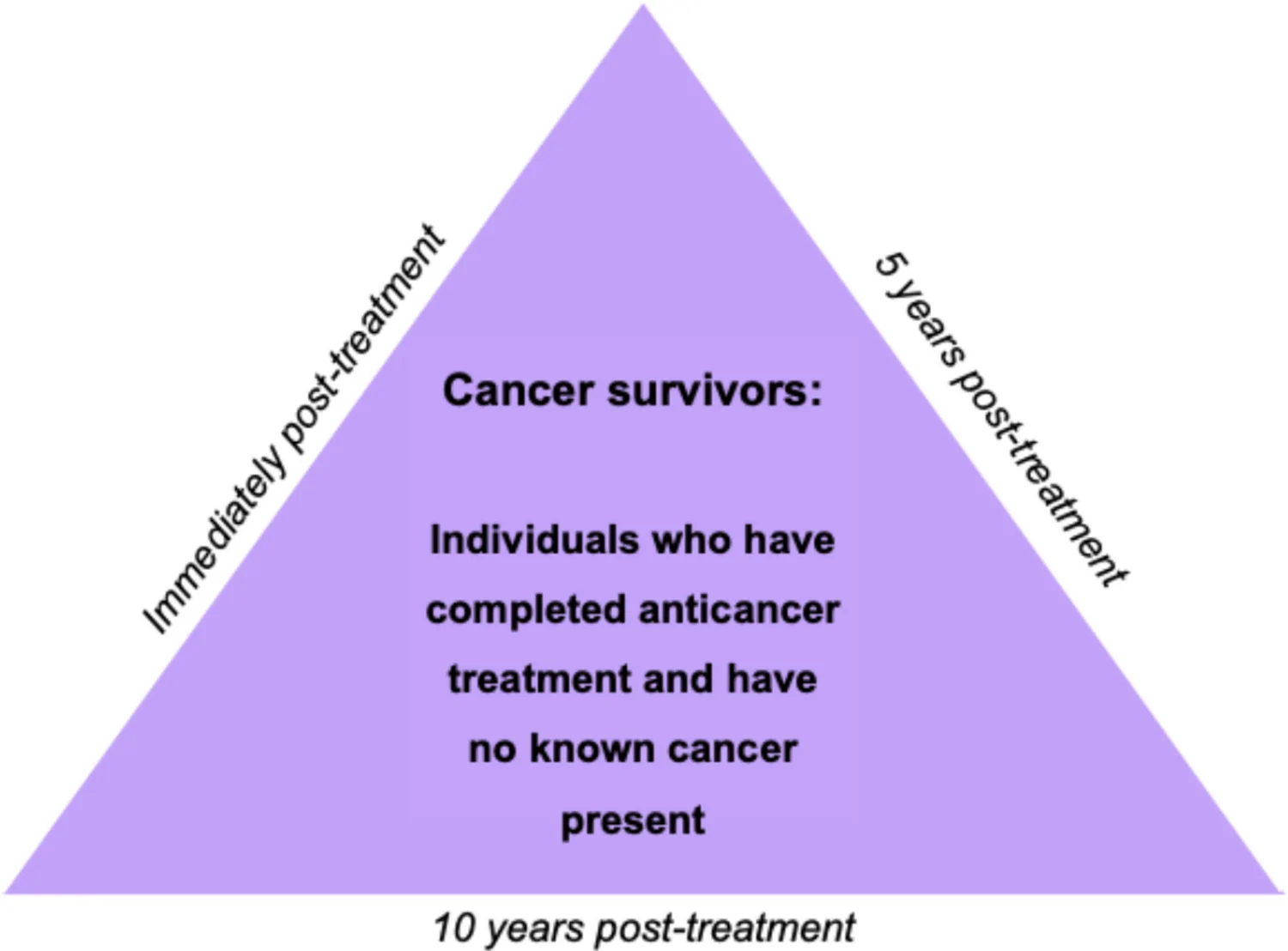
Consensus statements on who should be defined as “cancer survivors”

This e-Delphi process has reiterated ongoing issues/concerns around cancer survivor terminology among European specialist palliative care and oncology experts. Importantly, both expert groups agreed with (and preferred) ESMO’s functional definition of cancer survivors (individuals post curative-intent treatment) [3]. Indeed, both expert groups reached consensus agreement that individuals post-treatment with no evidence of disease (immediately, and after 5 and 10 years) should be called “cancer survivors” (Fig. 1), aligning with certain literature associated with the term [5]. Such lower agreement with the NCI definition [2] may reflect a lower acceptability of this predominantly North American interpretation outside of that area, as has been previously suggested [8].

Relatedly, there was also consensus in both expert groups that the term “cancer survivor” should not be applied to all individuals with cancer, and specifically not to individuals who are still receiving anticancer treatment with palliative intent, to individuals who have progressive/worsening cancer, and have no other anticancer treatment options, and to family members, friends, and caregivers of individuals with cancer or a history of cancer [30]. Nevertheless, certain learned groups (non-European in composition) do apply cancer survivor terminology to individuals with advanced or metastatic cancer [12], although it remains uncertain whether relevant people are supportive of these terms [31–33]. It should be noted that there was a general discomfort about the term “cancer survivor” among both expert groups, based upon free text feedback during the Delphi process. One participant stated, “I think to use cancer survivor to describe this group could be distressing and actually insulting”. Indeed, another found the very title of the study challenging (PRiSM, which referred to “cancer survivorship”), commenting, “since I disagree with this definition, it is difficult to answer questions (that relate to it).”

One of the persuasive arguments for not having an all-inclusive definition relates to the fact that people with cancer (or a history of cancer) are extremely heterogenous, and that while there are many generic problems, there are also highly specific problems relating to the individual’s cancer subtype, disease status, treatment history (current, previous), and their expected outcome (control, “cure”). Indeed, this explains the support from the expert groups for the broader living with, through, and beyond cancer categorisation [20], and equally the much broader ADPM categorisation. Participants even divided people on anticancer treatment between those on curative intent treatment, and those on palliative intent treatment, given the different anticipated trajectories. However, such clear identification of treatment intent may be considered challenging in the current era of novel anticancer treatments [34].

Consensus is not typically reached for all items in a Delphi study [35], resulting from variations between specialties, qualifications, personal experience/values, exposure to the issues [36, 37], and questionnaire structure/design [38]. The fact that English was not the first language for all participants may have been relevant, and indeed one participant commented that they felt their language limitation may have affected their ability to suggest alternative phrasing. Indeed, the very term “survivor” likely has different cultural and linguistic interpretations [8]. Additionally, the two expert groups differed in some important areas, e.g. the SPC group disagreed that “cancer survivor” should be applied to individuals who are still receiving anticancer treatment irrespective of treatment intent, whereas the ONC group only disagreed that “cancer survivor” should be applied to individuals who are still receiving anticancer treatment with palliative intent. There are no guidelines for when consensus is not achieved [25], although adjunctive methods such as the nominal group technique or consensus conferences could be considered [39, 40].

The strengths of this study are its transparent, robust methodology [22], with the survey developed from a detailed review of literature, while also encouraging anonymised participant modification to minimise potential biases [41, 42]. The expert samples and high completion rate were also strengths, incorporating a variety of geographical locations, healthcare disciplines, and non-professional backgrounds through the purposive sampling strategy [24, 43]. However, the panels were skewed towards representation from physicians and western Europe, and limited in representation from PPI/patient advocacy groups. Ultimately, these results represent one group of experts at one point in time [36], and potential differences according to geographic location or discipline within this group are unknown. Hence, additional research should corroborate these results [44]. Moreover, the opinions of people who have/had cancer should also be sought, particularly those on treatment, and those with advanced/metastatic cancer, especially across Europe, who have been underrepresented in previous research.

## Conclusion

European oncology and specialist palliative care experts demonstrated agreement that the term “cancer survivor” should be applied to people who have completed anticancer treatment and have no evidence of disease, and that application to people who have active cancer should be reconsidered. They also felt that pragmatic (factual) categories may be more useful in terms of providing tailored care for people who have/have had cancer.

## Supplementary Information

Below is the link to the electronic supplementary material.

Supplementary file1 (PDF 273 kb)

## Data Availability

The research data is available as supplementary information.
