# Supplementary material for: e-Delphi study on European expert views about “cancer survivor” and related terminologies
Source: J Cancer Surviv. 2026 Jan 31;20(Suppl 2):1479–88. doi: 10.1007/s11764-026-01972-z (PMC13744528; doi:10.1007/s11764-026-01972-z)
Supplement: Supplementary file 1 [file 11764_2026_1972_MOESM1_ESM.pdf]

Supplementary information

Title

e-Delphi study on European healthcare professionals' views about "cancer survivor" and related terminologies

Authors

Amy Taylor[1,2], Andrew Davies[1-3], PRiSM (Specialist Palliative Care's Role in Cancer Survivorship Model) e-Delphi Group

Affiliations

[1]School of Medicine, Trinity College Dublin, Ireland

[2]Academic Department of Palliative Medicine, Our Lady's Hospice & Care Services, Ireland

[3]School of Medicine, University College Dublin, Ireland

Corresponding author:

Dr Amy Taylor, Academic Department of Palliative Medicine, Our Lady's Hospice & Care Services, Harold's Cross, Dublin, D6W RY72, Ireland.

Email: tayloram@tcd.ie

Journal:

Journal of Cancer Survivorship

Table of Contents

Supplementary Tables S1a-b: Specialist palliative care (SPC) expert group results 3

S1a. Definition of “cancer survivor” – SPC group results 3

S1b .Categorisation of people who have/had cancer – SPC group results 6

Supplementary Tables S2a-b: Oncology (ONC) expert group results 7

S2a. Definition of “cancer survivor” – ONC group results 7

S2b. Categorisation of people who have/had cancer – SPC group results 10

Supplementary Table S3: Comparison of levels of agreement between SPC and ONC groups regarding final statements 11

Supplementary Tables S1a-b: Specialist palliative care (SPC) expert group results

S1a. Definition of "cancer survivor" – SPC group results

Statement	Round 1					Round 2					Round 3					rho	p value
	SD	MD	N	MA	SA	SD	MD	N	MA	SA	SD	MD	N	MA	SA		
**The following groups should be called cancer survivors:**																	
All individuals with cancer.	58%	15%	8%	7%	11%	69%	12%	7%	4%	8%						0.825**	<0.001
	73%		8%	18%		82%		7%	12%								
Individuals who are still receiving anticancer treatment.	42%	21%	8%	17%	12%												
	63%		8%	29%													
Individuals who have just completed anticancer treatment, and still have some known cancer present.	38%	23%	7%	19%	13%	44%	23%	7%	16%	9%						0.901**	<0.001
	61%		7%	32%		67%		7%	25%								
Individuals who have just completed anticancer treatment, and have no known cancer present.	14%	5%	12%	26%	43%	10%	5%	11%	19%	56%						0.849**	<0.001
	19%		12%	69%		15%		11%	75%								
Individuals who completed anticancer treatment at least 1 year beforehand, and have no known cancer present.	12%	8%	11%	20%	49%	11%	7%	9%	15%	58%						0.866**	<0.001
	20%		11%	69%		18%		9%	73%								
Individuals who completed anticancer treatment at least 5 years beforehand, and have no known cancer present.	6%	10%	5%	15%	64%	2%	2%	5%	12%	78%						0.684**	<0.001
	16%		5%	79%		4%		5%	90%								

Individuals who completed anticancer treatment at least 10 years beforehand, and have no known cancer present.	7%	6%	7%	12%	68%	2%	2%	6%	7%	81%						0.663**	<0.001
	13%		7%	80%		4%		6%	88%								
Individuals who have progressive/worsening cancer, and have no other anticancer treatment options.	68%	12%	7%	5%	8%	75%	12%	5%	4%	4%						0.775**	<0.001
	80%		7%	13%		87%		5%	8%								
Family members, friends, and caregivers of individuals with cancer or a history of cancer.	80%	10%	7%	2%	1%	89%	5%	5%	1%	0%						0.702**	<0.001
	90%		7%	3%		94%		5%	1%								
No individual should be called a “cancer survivor” i.e. the term should not be used.	25%	24%	32%	7%	12%	28%	17%	38%	7%	9%						0.912**	<0.001
	49%		32%	19%		45%		38%	16%								
Individuals who are still receiving anticancer treatment with curative intent						32%	28%	19%	12%	9%	51%	26%	11%	8%	4%	0.684	<0.001
						60%		19%	21%		77%		11%	12%			
Individuals who are still receiving anticancer treatment with palliative intent						58%	15%	16%	6%	5%	80%	5%	9%	5%	1%	0.626	<0.001
						73%		16%	11%		85%		9%	6%			

Key

SD	Strongly disagree	rho	Spearman’s rank correlation coefficient between relevant subsequent rounds
MD	Moderately disagree	*	Correlation is significant at the 0.05 level (2-tailed)
N	Neither agree nor disagree	**	Correlation is significant at the 0.01 level (2-tailed)

MA	Moderately agree	Shaded box	Consensus reached (≥75%)
SA	Strongly agree		
SPC	Specialist palliative care		
SPCS	Specialist palliative care services		

S1b. Categorisation of people who have/had cancer – SPC group results

**Statement**	**Round 1**					**Round 2**					**Round 3**					**rho**	**p value**
	**SD**	**MD**	**N**	**MA**	**SA**	**SD**	**MD**	**N**	**MA**	**SA**	**SD**	**MD**	**N**	**MA**	**SA**		
**The following classifications for groups of individuals with cancer or a history of cancer are useful:**																	
Seasons of survivorship	29%	25%	18%	21%	7%	38%	27%	20%	14%	1%						0.760**	<0.001
	54%		18%	28%		65%		20%	15%								
Living with, through and beyond cancer	2%	11%	8%	48%	31%	1%	9%	6%	56%	28%						0.937**	<0.001
	13%		8%	79%		10%		6%	84%								
New categorisation	6%	4%	7%	43%	40%	4%	2%	5%	52%	37%						0.932**	<0.001
	10%		7%	83%		6%		5%	89%								

Key

SD	Strongly disagree	rho	Spearman's rank correlation coefficient between relevant subsequent rounds
MD	Moderately disagree	*	Correlation is significant at the 0.05 level (2-tailed)
N	Neither agree nor disagree	**	Correlation is significant at the 0.01 level (2-tailed)
MA	Moderately agree	Shaded box	Consensus reached (≥75%)
SA	Strongly agree		
SPC	Specialist palliative care		
SPCS	Specialist palliative care services		

Supplementary Tables S2a-b: Oncology (ONC) expert group results

S2a. Definition of "cancer survivor" – ONC group results

Statement	Round 1					Round 2					Round 3					rho	p value
	SD	MD	N	MA	SA	SD	MD	N	MA	SA	SD	MD	N	MA	SA		
**Section 2: Definition of "cancer survivor"**																	
**The following groups should be called cancer survivors:**																	
All individuals with cancer.	45%	26%	9%	4%	15%	52%	27%	8%	4%	10%						0.907**	<0.001
	71%		9%	19%		79%		8%	14%								
Individuals who are still receiving anticancer treatment.	21%	25%	19%	21%	15%												
	46%		19%	36%													
Individuals who have just completed anticancer treatment, and still have some known cancer present.	21%	26%	19%	17%	17%	21%	37%	12%	19%	12%						0.960**	<0.001
	47%		19%	34%		58%		12%	31%								
Individuals who have just completed anticancer treatment, and have no known cancer present.	8%	9%	8%	15%	60%	6%	6%	6%	21%	62%						0.963**	<0.001
	17%		8%	75%		12%		6%	83%								
Individuals who completed anticancer treatment at least 1 year beforehand, and have no known cancer present.	9%	9%	4%	25%	53%	6%	4%	2%	27%	62%						0.788**	<0.001
	18%		4%	78%		10%		2%	89%								
Individuals who completed anticancer treatment at least 5 years beforehand, and have no known cancer present.	8%	6%	2%	11%	74%	2%	2%	2%	8%	87%						0.657**	<0.001
	14%		2%	85%		4%		2%	95%								

Individuals who completed anticancer treatment at least 10 years beforehand, and have no known cancer present.	8%	8%	2%	15%	68%	0%	2%	2%	13%	83%						0.590**	<0.001
	16%		2%	83%		2%		2%	96%								
Individuals who have progressive/worsening cancer, and have no other anticancer treatment options.	51%	30%	6%	6%	8%	62%	25%	2%	4%	8%						0.834**	<0.001
	81%		6%	14%		87%		2%	12%								
Family members, friends, and caregivers of individuals with cancer or a history of cancer.	66%	17%	11%	4%	2%	77%	12%	8%	2%	2%						0.770**	<0.001
	83%		11%	6%		89%		8%	4%								
No individual should be called a “cancer survivor” i.e. the term should not be used.	51%	13%	17%	11%	8%	56%	13%	17%	12%	2%						0.915**	<0.001
	64%		17%	19%		69%		17%	14%								
Individuals who are still receiving anticancer treatment with curative intent						17%	31%	13%	23%	15%	18%	35%	4%	31%	12%	0.894**	<0.001
						48%		13%	38%		53%		4%	43%			
Individuals who are still receiving anticancer treatment with palliative intent						50%	21%	8%	12%	10%	63%	14%	2%	12%	8%	0.887**	<0.001
						71%		8%	22%		77%		2%	20%			

Key

SD	Strongly disagree	rho	Spearman’s rank correlation coefficient between relevant subsequent rounds
MD	Moderately disagree	*	Correlation is significant at the 0.05 level (2-tailed)
N	Neither agree nor disagree	**	Correlation is significant at the 0.01 level (2-tailed)

MA	Moderately agree	Shaded box	Consensus reached (≥75%)
SA	Strongly agree		
SPC	Specialist palliative care		
SPCS	Specialist palliative care services		

S2b. Categorisation of people who have/had cancer – ONC group results

Statement	Round 1					Round 2					Round 3					rho	p value
	SD	MD	N	MA	SA	SD	MD	N	MA	SA	SD	MD	N	MA	SA		
**The following classifications for groups of individuals with cancer or a history of cancer are useful:**																	
Seasons of survivorship	17%	23%	15%	32%	13%	19%	21%	13%	35%	12%						0.908**	<0.001
	40%		15%	43%		40%		13%	47%								
Living with, through and beyond cancer	6%	8%	4%	49%	34%	6%	2%	2%	58%	33%						0.898**	<0.001
	14%		4%	83%		8%		2%	91%								
New categorisation	2%	9%	15%	42%	32%	2%	10%	10%	50%	29%						0.886**	<0.001
	11%		15%	74%		12%		10%	79%								

Key

SD	Strongly disagree	rho	Spearman’s rank correlation coefficient between relevant subsequent rounds
MD	Moderately disagree	*	Correlation is significant at the 0.05 level (2-tailed)
N	Neither agree nor disagree	**	Correlation is significant at the 0.01 level (2-tailed)
MA	Moderately agree	Shaded box	Consensus reached (≥75%)
SA	Strongly agree		
SPC	Specialist palliative care		
SPCS	Specialist palliative care services		

Supplementary Table S3: Comparison of levels of agreement between SPC and ONC groups regarding final statements

Statement	$x^2(1)$	p value	n
**Section: Definition of "cancer survivor"**			
**The following groups should be called "cancer survivors":**			
All individuals with cancer.	0.294	0.588	52
Individuals who have just completed anticancer treatment, and still have some known cancer present.	1.030	0.310	52
Individuals who have just completed anticancer treatment, and have no known cancer present.	2.000	0.157	52
Individuals who completed anticancer treatment at least 1 year beforehand, and have no known cancer present.	6.441	***0.011***	52
Individuals who completed anticancer treatment at least 5 years beforehand, and have no known cancer present.	0.995	0.319	52
Individuals who completed anticancer treatment at least 10 years beforehand, and have no known cancer present.	2.773	0.096	52
Individuals who have progressive/worsening cancer, and have no other anticancer treatment options.	1.300	0.254	52
Family members, friends, and caregivers of individuals with cancer or a history of cancer.	3.071	0.080	52
No individual should be called a "cancer survivor" i.e. the term should not be used.	0.249	0.618	52

Individuals who are still receiving anticancer treatment with curative intent	49.133	***<0.001***	49
Individuals who are still receiving anticancer treatment with palliative intent	16.641	***<0.001***	49
**Section: Categorisation of individuals with cancer or a history of cancer**			
**The following classifications for groups of individuals with cancer or a history of cancer are useful:**			
Seasons of survivorship	40.540	***<0.001***	52
Living with, through and beyond cancer	1.577	0.209	52
New categorisation	5.327	***0.021***	52

DOF=1 for all statements

For a significance level of 0.05 and one degree of freedom, the critical value is 3.841.

Key:

SPC = specialist palliative care expert group; ONC = oncology expert group

SPCS = specialist palliative care services

$x^2(1)$ = Chi square test for goodness of fit comparing the levels of agreement of the ONC group with the SPC group for each statement, for one degree of freedom

N/A = unable to perform x^2 test as at least one group achieved 100% agree / not agree and comparison is not possible

n = number of participants in ONC expert comparison group who responded to the statement

Italicised, bold p value = significant ($p<0.05$)

Pale grey box = Statement achieved SPC consensus but not ONC consensus

Dark grey box = Statement achieved ONC consensus but not SPC consensus
